# A Cheap and Expeditious Way of Saturating the Aq. Amm. Acet. with Carbonic Acid Gas

**Published:** 1799-10

**Authors:** 


### *A Cheap and expeditious way of saturating the Aq. Amm. Acet. with Carbonic Acid Gas.*

Take a common stopper-bottle, the one which it is usually kept in the shop, and fill it about two thirds with acetum distillatum; then weigh the requisite proportion of ammonia, which break into lumps of a size sufficient to be admitted into the bottle, and put them in directly one after another; as, if the ammonia is broke too small, or put in too suddenly, it occasions too quick an extrication of the gas, and a quantity of it is lost. The stopper of the bottle must then be tied over with a piece of leather, and put in its usual place; interposing a substance between the top of the bottle and the superincumbent shelf, so as to fit tight, which considerably adds to the pressure, and tends to combine more intimately the carbonic acid gas; after having stood a few hours, the ammonia is dissolved, and the carbonic acid absorbed by the liquor.

The aqua ammoniæ acetata, thus prepared, is very strongly impregnated with carbonic acid gas; and is greatly deprived of that mawkish disagreeable taste which it has, when made in the usual way.

With respect to the properties of the aq. ammon. acet. as a medicine, it is unnecessary to particularize them, as they are so well known. The writer can, from experience, speak of its superior good effects as a febrifuge made as above, combined with carbonic acid gas, with this peculiar advantage, that it tends to keep the bowels open, even when under the influence of opiates.

It likewise sits easy upon most weak and irritable stomachs, when scarcely any other medicine would be retained; and as such might be used with propriety, in place of the saline draught, in a state of effervescence.

Its use as an external application has been often tried, with marked good effect, made in this manner; and from analogy I conceive it might be adopted with great advantage in a variety of cases, the application of which must be determined by the practitioners.

According to Bergman, ammonia contains $\frac{45}{100}$ of carbonic acid, $\frac{43}{100}$ of pure ammonia, $\frac{12}{100}$ of water, that is nearly half its weight of air; so that in a pint of the aq. ammon. acet. in which 4 drachms of ammonia is used, there are about 108 grains weight of air, which, according to its specific gravity, will be equivalent to $159\frac{1}{10}$ cubic inches of carbonic acid, the greater part of which unites with the liquor; so that the materials made use of for one pint (without any expence) are capable of furnishing more than four times their their bulk of carbonic acid, a quantity equal to any good effect, where it may be deemed useful to the stomach and bowels.

The intimate knowledge the writer has of the best manner of making the nephritic alkaline waters, first suggested to him the idea of combining the carbonic acid extricated from the ammonia, with the liquor itself, conceiving that it might be applied to some useful purpose. The nephritic alkaline waters, of themselves, are a sufficient proof of the antiseptic and good effects the carbonic acid has upon the animal economy; which waters, the writer has brought to the highest degree of perfection ever attained in this or any other country, by a machine, whose mechanical powers of pressure and retention cannot be exceeded; in which state he begs, through the medium of your valuable publication, to apprize the faculty, that the waters may be obtained, at his house in the city, with any proportion of alkali, as may be best suited to the peculiar circumstances of a patient's case.

Artificial Seltzer water, and other medicated waters, prepared upon the same principle, which are infinitely superior to the natural ones; containing more air, as well as a more select quantity of ingredients. It is unnecessary to particularize the virtues of the above waters, as no practitioner in medicine can be unacquainted with their high and valuable qualities.

